# GhWRKY33 Interacts with GhTIFY10A to Synergistically Modulate Both Ageing and JA-Mediated Leaf Senescence in *Arabidopsis*

**DOI:** 10.3390/cells11152328

**Published:** 2022-07-29

**Authors:** Songguo Wu, Huimin Zhang, Ruling Wang, Guimei Chang, Yifen Jing, Zhifang Li, Ligang Chen

**Affiliations:** 1 CAS Key Laboratory of Tropical Plant Resources and Sustainable Use, Xishuangbanna Tropical Botanical Garden, Chinese Academy of Sciences, Mengla 666303, China; wusongguo@xtbg.ac.cn (S.W.); zhanghuimin@xtbg.ac.cn (H.Z.); wrl@xtbg.org.cn (R.W.); changguimei@xtbg.ac.cn (G.C.); jingyifen@xtbg.ac.cn (Y.J.); 2 University of Chinese Academy of Sciences, Beijing 100049, China; 3 Center of Economic Botany, Core Botanical Gardens, Chinese Academy of Sciences, Mengla 666303, China; 4 School of Life Sciences, Henan University, Kaifeng 475004, China; 10140165@vip.henu.edu.cn

**Keywords:** *GhWRKY33*, jasmonate acid, *GhTIFY10* *A*, leaf senescence

## Abstract

WRKY transcription factors play critical roles in the modulation of transcriptional changes during leaf senescence, but the underlying mechanisms controlled by them in this progress still remain enigmatic. In this study, *Gossypium hirsutum* WRKY DNA-binding protein 33 (*GhWRKY33*) was characterized as a negative regulator of both ageing and JA-mediated leaf senescence. The overexpression of *GhWRKY33* in *Arabidopsis* greatly delayed leaf senescence, as determined by elevated chlorophyll content, lower H_2_O_2_ content, and reduced expression of several senescence-associated genes (*SAGs*). An electrophoretic mobility shift assay (EMSA) and transient dual–luciferase reporter assay revealed that GhWRKY33 could bind to the promoters of both *AtSAG12* and *Ghcysp* and suppress their expression. Yeast two-hybrid (Y2H) and firefly luciferase complementation imaging (LUC) assays showed that GhWRKY33 could interact with GhTIFY10A. Similarly, the overexpression of *GhTIFY10A* in *Arabidopsis* also dramatically delayed leaf senescence. Furthermore, both *GhWRKY33* and *GhTIFY10A* negatively regulate JA-mediated leaf senescence. In addition, a transientdual-luciferase reporter assay indicated that GhWRKY33 and GhTIFY10A could function synergistically to inhibit the expression of both *AtSAG12* and *Ghcysp*. Thus, our work suggested that *GhWRKY33* may function as a negative regulator to modulate both ageing and JA-mediated leaf senescence and also contributes to a basis for further functional studies on cotton leaf senescence.

## 1. Introduction

Plant senescence causes a series of active degenerative alterations at the cellular, tissue, organ, and organism levels and often accompanies color changes and the shedding of leaves in autumn. At the growth and maturation stages, leaves are the primary photosynthetic organ for energy harvesting and nutrient production. When a leaf initiates senescing, it then serves as a source for mobilizable nutrients to increase reproductive success [1]. Leaf senescence is a programmed cell death process controlled by a highly regulated genetic network. This process represents one of the external manifestations of plant growth and development in response to the adverse environment. Normally, plant senescence contributes to survival under various adverse environmental conditions. Senescence is normally initiated in an ageing-dependent manner, but environmental signals and multiple phytohormones can also trigger it. Several phytohormones, such as jasmonic acid (JA), abscisic acid (ABA), ethylene (ET), salicylic acid (SA), and gibberellin (GA), have been suggested to promote leaf senescence through complex interconnecting pathways [2,3,4].

Numerous studies have demonstrated that JA plays an important role in leaf senescence through the regulation of various senescence-associated genes in several plant species, such as *Zea mays*, *Oryza sativa*, and *Arabidopsis thaliana* [1,5,6,7]. The exogenous application of JA or an increase of endogenous JA content promotes leaf senescence, while the repression of JA biosynthesis delays this process [8,9]. More importantly, key components in JA signaling are also involved in plant senescence regulation. For example, mutation of *COI1* or overexpression of *JAZ1△3A* in *Arabidopsis* blocked JA-induced leaf senescence and exhibited a stay-green phenotype [10]. The bHLH family subfamily IIIe transcription factors *(AtMYC2*, *AtMYC3*, and *AtMYC4*) and the bHLH family subfamily IIId transcription factors (*AtbHLH03*, *AtbHLH13*, *AtbHLH14*, and *AtbHLH17*) antagonize each other and regulate the expression of downstream senescence-associated genes (*SAG*s), thus regulating JA-induced leaf senescence [10]. Another study also showed that AtMYC2 can directly repress *AtCAT2* expression and promote JA-induced hydrogen peroxide accumulation and leaf senescence [11].

The orderly physiological and biochemical changes of leaves during senescence are often closely associated with the expression changes of numerous *SAG*s. Interestingly, many members of the WRKY family genes were found to express strongly in senescing leaves, and they ranked second in the senescence-associated transcription factor families based on senescence transcriptome [12], supporting their involvement in leaf senescence. Growing evidence has shown that WRKY proteins function as critical components in senescence-associated regulatory pathways. Both AtWRKY45 and AtWRKY75 can interact with DELLA proteins to modulate GA-mediated leaf senescence [4,13,14]. AtWRKY57 can form complexes with JASMONATE ZIM-DOMAIN4/8 (AtJAZ4/8) and the AUX/IAA protein AtIAA29 to mediate the crosstalk between JA- and auxin-mediated signaling pathways in JA-induced leaf senescence [3]. Recently, several cotton WRKY TFs have also been reported to play a role in leaf senescence. For example, overexpression of *GhWRKY27* or *GhWRKY42* in *Arabidopsis* can promote leaf senescence, while overexpression of *GhWRKY91* in *Arabidopsis* delayed this process [15,16,17]. However, it is still unclear whether cotton WRKY proteins can interact with certain phytohormones and function together to modulate leaf senescence.

As an important economic crop, cotton acts as the dominant raw material for the textile industry. The earliness of cotton severely restricts the yield and quality of cotton fiber, dramatically affecting the processing and production of cotton products [18]. The identification of critical senescence-associated genes may contribute to the genetic breeding of excellent cotton varieties. In the present study, we performed molecular and genetic assays to elucidate the molecular function of *GhWRKY33* in both ageing and JA-triggered leaf senescence. The overexpression of *GhWRKY33* in *Arabidopsis* delayed leaf senescence by directly binding to the promoters of *SAG*s, such as *AtSAG12* and *Ghcysp*, and suppressing their expression. In addition, GhWRKY33 can physically interact with GhTIFY10A. Consistent with *GhWRKY33*, overexpression of *GhTIFY10A* in *Arabidopsis* also delayed leaf senescence. Furthermore, both *GhWRKY33* and *GhTIFY10A* can delay JA-induced leaf senescence, and they function synergistically to repress the expression of both *AtSAG12* and *Ghcysp*. Therefore, *GhWRKY33* may function as a potential ageing-related factor to regulate both ageing and JA-induced leaf senescence, and our studies also provide new mechanistic insight into the roles of *WRKY* genes in leaf senescence in cotton.

## 2. Materials and Methods

### 2.1. Growth of Plant Materials

The *G. hirsutum* cultivar, Zhongzhimian 2, plants were grown in an artificial growth chamber at 28 °C under LDs (16 h light/8 h dark cycle), and the *Arabidopsis* plants were grown in an artificial growth chamber at 22 °C under LDs (16 h light/8 h dark cycle).

### 2.2. Generation of Transgenic Overexpression Lines

To generate the *35S:GhWRKY33* and *35S:GhTIFY10A*, the cDNA fragments containing the full coding sequence were cloned into the same restriction sites of the *Agrobacterium* transformation vector *pOCA30* in the sense orientation driven by the CaMV 35S promoter. The floral dip procedure performed the *Arabidopsis* transformation. The seeds were collected from transformed plants and selected on 1/2 Murashige & Skoog medium containing 50 μg/mL kanamycin. Kanamycin-resistant plants were transferred to soil 8 d after germination and were grown in a growth chamber. The primers used for identifying transgenic overexpression lines are listed in Table S1.

### 2.3. Gene Expression Analysis

For RT–qPCR analysis, total RNA was extracted using TRIzol reagent (Invitrogen, Carlsbad, CA, USA) and was treated with RNase-free DNase, according to the manufacturer’s instructions. Total RNA (1 μg) was reverse-transcribed in a 20 μL reaction mixture using Superscript II (Invitrogen, Carlsbad, CA, USA). After the reaction, 1μL aliquots were used as templates for RT-qPCR. Half-reactions (10 μL each) were performed with the Light Cycler FastStart DNA Master SYBR Green I Kit on a Light Cycler 480 real-time PCR machine (Roche, Mannheim, Germany), according to the manufacturer’s instructions. *AtActin2* and *GhActin* were used as controls in RT–qPCR [15,19]. Analysis was conducted following the minimum information for publication of quantitative Real-Time PCR experiments guidelines [20]. Primers used for RT–qPCR analysis are listed in Table S1. 

### 2.4. EMSA Assays

The full-length *GhWRKY33* CDS was cloned into *pGEX-4T-1*. All plasmids were introduced into *Escherichia coli* BL21 cells, and Glutathione S-transferase (GST), GST-GhWRKY33 protein expression was induced by 0.5 mM Isopropyl β-D-1-thiogalactopyranoside for 24 h at 16 °C. Soluble GST and GST-GhWRKY33 were extracted and immobilized to glutathione beads (Thermo Fisher Scientific, Waltham, MA, USA). The purified GST-GhWRKY33 protein was confirmed by SDS-PAGE and used for EMSA. The EMSA assay was conducted using a Chemiluminescent EMSA Kit (Beyotime) following the manufacturer’s protocol. The DNA fragments of the *AtSAG12* or *Ghcysp* promoter were synthesized, and biotin was labeled to the 5′terminal of DNA at Beijing Genomics Institute (Beijing, China). Biotin-unlabeled fragments of the same sequences or mutated sequences were used as competitors, and the GST protein alone was used as the negative control.

### 2.5. Yeast Two-Hybrid Screening and Confirmation

The full-length CDS of *GhWRKY33* was cloned into the bait vector *pGBKT7* and then transformed into the yeast strain Y_2_HGold (Clontech, Mountain View, CA, USA). The two-hybrid screening was performed via the mating protocol described in Clontech’s Matchmaker Gold Yeast Two-Hybrid user manual. To confirm protein–protein interactions, the full-length CDS of *GhTIFY10A* were cloned into the prey vector *pGADT7*.

### 2.6. Luciferase Complementation Imaging Assay (LCI)

The full-length CDS of *GhWRKY33* and *GhTIFY10A* were fused with *pCAMBIA1300-cLUC* and *pCAMBIA1300-nLUC*, respectively. Then, the assays were performed as described previously [21]. 

### 2.7. Leaf Senescence Assays

Leaves from four-week-old plants were used for the JA-induced leaf senescence assay. The detached leaves were placed into dishes filled with distilled water or water with 100 µM MeJA and then kept in weak light (20 µmol m^−2^ s^−1^ photosynthetic photon flux density) at 22 °C. Chlorophyll was then extracted with 80% acetone from detached leaves, and its content was determined at 663 and 645 nm, according to Lichtenthaler (1987) [22]. Membrane ion leakage was determined as described in Jiang et al., 2007 [23]. The H_2_O_2_ content was measured with the H_2_O_2_ content detection Kit (BC3595, Solarbio, Beijing, China) using the titanium sulfate colorimetric method. 

### 2.8. Transient Expression Assay

The promoter regions of both *AtSAG12* and *Ghcysp* were amplified and cloned into the *pGreenII 0800-LUC* vector as reporters. Full-length CDS of *GhWRKY33*, *GhTIFY10A,* and *GFP* were amplified and cloned into the *pGreenII 62-SK* vector as effectors. Then the transient expression assay was performed according to Wang et al., 2016 [24]. *Agrobacterium tumefaciens* GV3101 harboring the above constructs was infiltrated into five-week-old *Nicotiana benthamiana* leaves using a needleless syringe for transactivation analyses. After growing for 48 h under the condition of 16 h of light and 8 h of dark, leaves were injected with 0.94 mM luciferin, and the resulting luciferase signals were captured using the Tanon-5200 image system. These experiments were repeated at least three times with similar results. Quantitative analysis was performed using ImageJ software.

## 3. Results

### 3.1. Overexpression of GhWRKY33 Delayed Leaf Senescence in Transgenic Arabidopsis Plants

**Figure 1:**
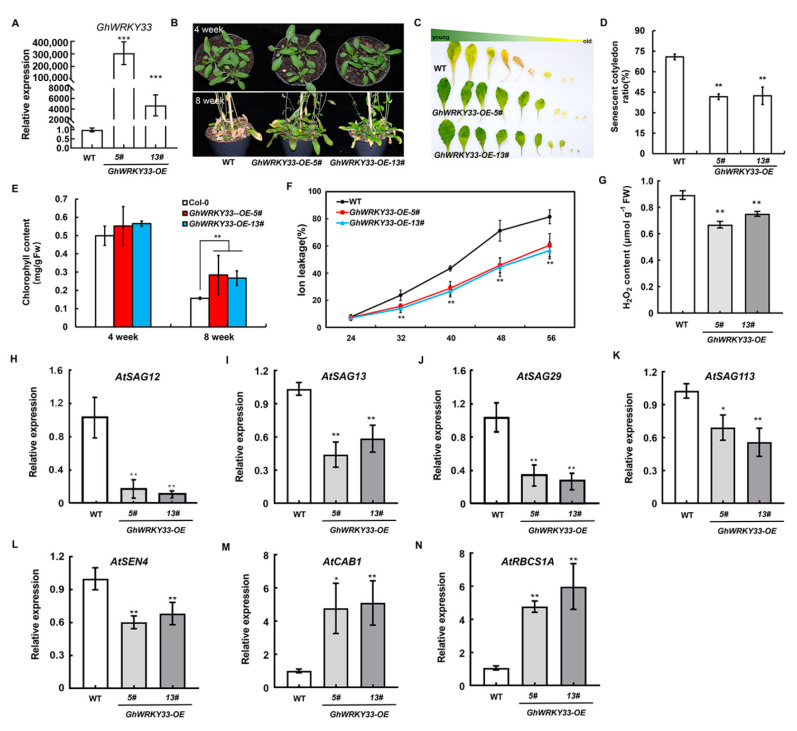
Overexpression of *GhWRKY33* in *Arabidopsis* delayed leaf senescence. (**A**) Transcript levels of *GhWRKY33* in transgenic plants. (**B**) Phenotypic characteristics of WT and transgenic plants grown for four and eight weeks, respectively. Plants were grown in a growth chamber at 22 °C under a 16 h light/8 h dark photoperiod, and the natural senescence phenotype was observed from about 72 plants for each line, and representative plants were photographed. (**C**) Senescence symptoms of detached leaves of eight-week-old WT and transgenic plants sorted based on their age. (**D**) Relative senescent cotyledon ratio of WT and *GhWRKY33-OE* plants grown for four weeks. (**E**) Chlorophyll content in detached rosette leaves of WT and *GhWRKY33-OE* plants grown for four and eight weeks, respectively. FW, fresh weight. (**F**) Membrane ion leakage of WT and *GhWRKY33*-OE plants at the indicated leaf age. Leaves three to four from 12 to 15 plants of each genotype (approximately 26 leaves) were harvested and pooled. (**G**) H_2_O_2_ content in rosette leaves of WT and *GhWRKY33-OE* plants grown for eight weeks. (**H**–**N**) Transcript levels of *AtSAGs, AtCAB1,* and *AtRBCS1A* in the indicated genotypes. Data from three biological replicates were analyzed by ANOVA, and asterisks show significant differences compared with WT (* *p* < 0.05; ** *p* < 0.01; *** *p* < 0.001).

During our studies about the role of GhWRKY members, we found that *GhWRKY33* may act as a potential candidate that plays a role in leaf senescence. Then, to confirm the role of *GhWRKY33* in leaf senescence, four homozygous transgenic *Arabidopsis* lines (T_3_) heterologously expressing *GhWRKY33* under the control of the CaMV 35S promoter (*GhWRKY33-OE-3*#, *GhWRKY33-OE-5*#, *GhWRKY33-OE-13*# and *GhWRKY33-OE-16*#) were used for further study (Figure 1A and Figure S1A). We observed that the four lines all showed a similar phenotype to wild-type in terms of overall development and flowering time. Interestingly, ageing-triggered leaf senescence was delayed in *GhWRKY33*-overexpressing plants compared with wild-type in a dose-independent manner of *GhWRKY33* (Figure 1B–D and Figure S1B). Transgenic plants can survive about 15 more days than wild-type plants, on average. The transgenic plants also displayed a significantly elevated chlorophyll content, lower ion leakage, significantly reduced H_2_O_2_ content, and reduced expression of several *SAGs* but the enhanced expression of the photosynthetic genes (*AtCAB1* and *AtRBCS1A*) than wild-type plants (Figure 1E–N). Thus, the constitutive overexpression of *GhWRKY33* in *Arabidopsis* led to delayed leaf senescence.

### 3.2. GhWRKY33 Was Repressed in Senescing Leaves

**Figure 2:**
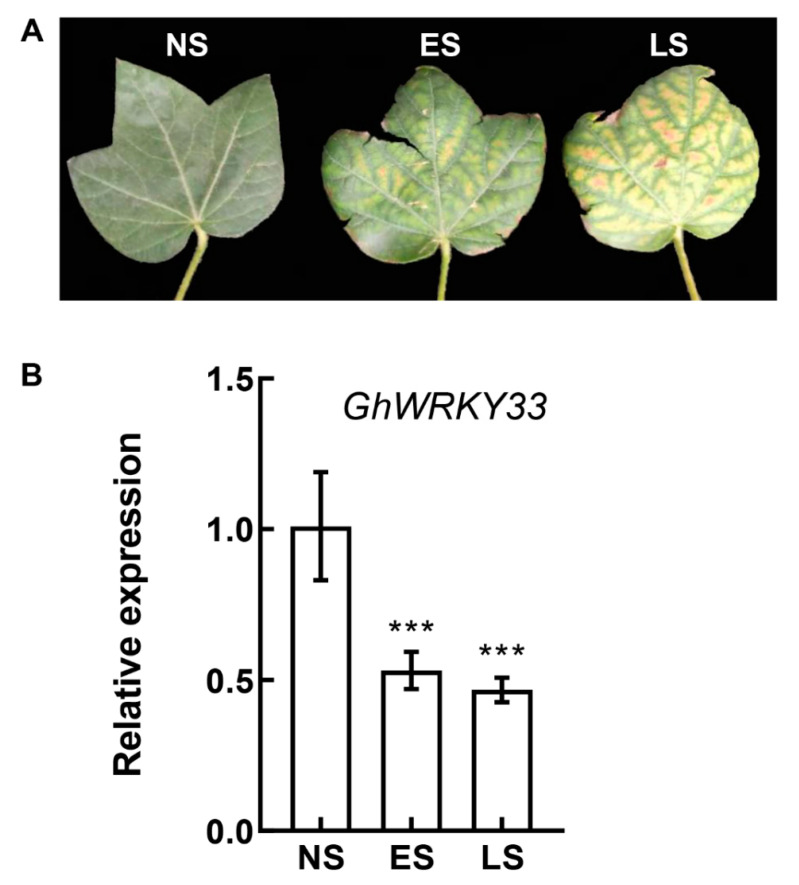
Expression of *GhWRKY33* in senescing leaves. (**A**) Different stages of leaf senescence. NS, a fully expanded, non-senescent leaf; ES, an early senescent leaf, with <50% leaf area yellowing; LS, a late senescent leaf, with >50% leaf area yellowing. (**B**) RT-qPCR analysis of *GhWRKY33* transcript levels in wild-type leaves at different developmental stages. Transcript levels of *GhWRKY33* in NS leaves were arbitrarily set to 1. Data from three biological replicates were analyzed by ANOVA, and asterisks indicate significant differences compared with NS leaves (*** *p* < 0.001).

Since *GhWRKY33* appears to act as a negative regulator in leaf senescence, we speculate that *GhWRKY33* may show altered expression in cotton senescing leaves. Then cotton leaves at different senescence stages were collected, and the expression pattern of *GhWRKY33* in these leaves was analyzed in Zhongzhimian 2 (Figure 2A). As shown in Figure 2B, compared with non-senescent leaves (NS), the expression level of *GhWRKY33* was dramatically lower in early senescent leaves (ES). It was further repressed during the late senescent leaves (LS). The results showed that the accumulation of *GhWRKY33* transcript was reduced during cotton leaf senescence. Based on Plant Public RNA-seq Database (PPRD, http://ipf.sustech.edu.cn/pub/plantrna/, accessed on 22 June 2022), we also observed repressed expression of *GhWRKY33* in senescing leaves in both TX2094 and Lumianyan 28 (Figure S2) [25]. Furthermore, as an orthologous gene of *GhWRKY33, AtWRKY70* has been demonstrated to function as a negative regulator of leaf senescence in *Arabidopsis thaliana* [26,27]. Thus, the results indicated that *GhWRKY33* may play a role in leaf senescence. 

### 3.3. GhWRKY33 Binds Directly to the Promoters of SAGs and Suppresses Their Expression

**Figure 3:**
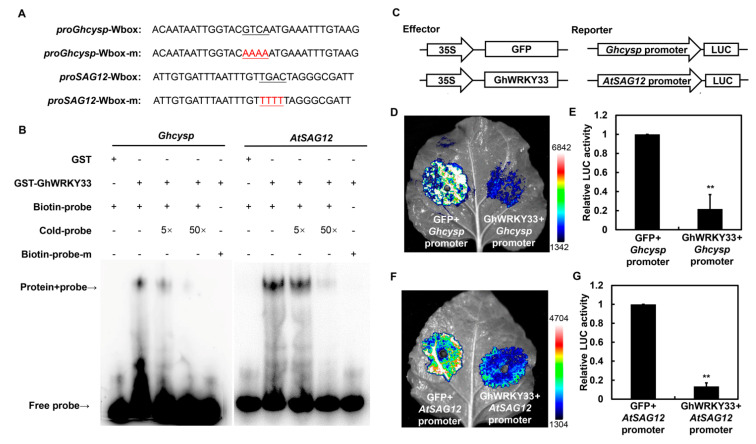
GhWRKY33 binds directly to the promoters of *SAGs* and suppresses their expression. (**A**) Probe sequences of *Ghcysp* and *AtSAG12* for the EMSA assays. (**B**) Binding of *GhWRKY33* to the W-boxes in the promoters of *Ghcysp* and *AtSAG12* in EMSA assays. “–” indicates absence, while “+” indicates presence. (**C**) Structure of reporter and effectors used in transient dual-luciferase reporter system. (**D**,**E**) GhWRKY33 suppresses *Ghcysp* expression in a transient dual-luciferase reporter system. The LUC/REN ratio of the combination of pGreenII-62-SK empty vector and *Ghcysp* was set as 1. (**F**,**G**) GhWRKY33 suppresses *AtSAG12* expression in transient dual-luciferase reporter system. The relative LUC activity of the combination of pGreenII-62-SK empty vector and *AtSAG12* was set as 1. Data from six biological replicates were analyzed by ANOVA, and asterisks indicate significant differences (** *p* < 0.01).

The WRKY TFs participated in various physiological processes by specifically binding to W-boxes (T/CTGACC/T) in the promoters of their target genes [28,29]. The above results showed that constitutive overexpression of *GhWRKY33* in *Arabidopsis* can delay leaf senescence. Therefore, we speculated that *GhWRKY33* might participate in leaf senescence by regulating the expression of *SAG*s. To test this hypothesis, we then analyzed the promoter sequence of *Ghcysp* and its homologue gene, *AtSAG12*, in *Arabidopsis*. Interestingly, W-box elements were found in the promoter sequence of both *Ghcysp* and *AtSAG12*, suggesting that *GhWRKY33* may directly regulate their expression during leaf senescence. Then, we performed EMSAs with the GST-GhWRKY33 recombinant protein to determine the in vitro binding of GhWRKY33 to both *Ghcysp* and *AtSAG12* promoters (Figure 3A). As shown in Figure 3B, GhWRKY33 could bind the probes containing the W-box sequence. The binding signals decreased after the addition of unlabeled WT competitors. In contrast, the GhWRKY33 protein did not bind to the mutant probe carrying a mutated W-box (Figure 3B). The GST protein alone also did not bind to both *Ghcysp* and *AtSAG12* promoters (Figure 3B).

These data suggest that GhWRKY33 may directly bind to the promoters of both *Ghcysp* and *AtSAG12* to modulate leaf senescence.

To further elucidate the direct regulation *GhWRKY33* on the expression of both *Ghcysp* and *AtSAG12*, a transient dual-luciferase assay was conducted in *Nicotiana benthamiana* leaves. Then both the promoters of *Ghcysp* and *AtSAG12* were fused with *LUC* gene as reporters (*Ghcysp:LUC* and *AtSAG12:LUC*) (Figure 3C). At the same time, the full-length CDS of *GhWRKY33* was driven by the CaMV35S promoter as an effector (Figure 3C). Co-expression of a reporter with effector plasmid in *N. benthamiana* leaves led to the repression of LUC compared with the control (Figure 3D–G and Figure S3). This result indicates that GhWRKY33 can inhibit the expression of both *Ghcysp* and *AtSAG12*.

### 3.4. GhWRKY33 Physically Interacts with GhTIFY10A

**Figure 4:**
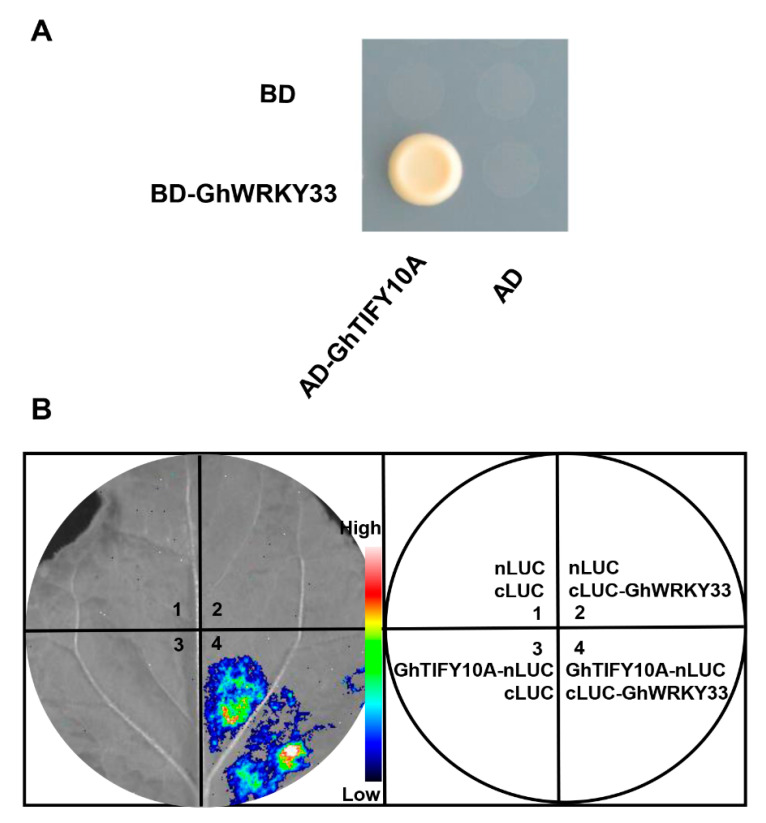
GhWRKY33 physically interacts with GhTIFY10A. (**A**) Yeast-two-hybrid assays. The interaction was indicated by the ability of cells to grow on a synthetic dropout medium lacking Leu/Trp/His/Ade. (**B**) GhWRKY33 associates with GhTIFY10A in LCI assays. LUC images of *N. benthamiana* leaves coinfiltrated with various constructs are shown in the lower quadrant of the circle. The pseudocolor bar shows the range of luminescence intensity.

Increasing evidence suggests that WRKY proteins function by forming protein complexes with other interactors [29]. We employed the yeast two-hybrid system to search for potential partners of the GhWRKY33 protein. *GhWRKY33* was fused with the BD domain of the pGBKT7 vector as bait. Yeast cells harboring the bait were transformed with a specific library containing GhTIFYs inserts for prey proteins fused to GAL4-AD. We found that GhWRKY33 can interact with GhTIFY6B and GhTIFY10A (Figure 4A and Figure S4). In this study, we pay attention to the possible role of *GhTIFY10A* in leaf senescence. GhTIFY10A contained both TIFY- and Jas-conserved domains and belongs to the JAZ subfamily of the TIFY protein family (Figure S5).

To determine whether GhWRKY33 interacts with GhTIFY10A in planta, we conducted firefly luciferase (LUC) complementation imaging (LCI) assays in *N. benthamiana* leaves. In these experiments, *GhWRKY33* was fused to the C-terminal half of LUC (cLUC) to produce GhWRKY33-cLUC, whereas GhTIFY10A was fused to the N-terminal half of LUC (nLUC) to produce nLUC-GhTIFY10A. *N. benthamiana* cells co-expressing GhWRKY33-cLUC and nLUC-GhTIFY10A displayed strong luminescence signals, whereas those co-expressing nLUC and GhWRKY33-cLUC or nLUC-GhTIFY10A and cLUC displayed no signal, confirming that the GhWRKY33-GhTIFY10A interaction occurs in vivo (Figure 4B).

### 3.5. Overexpression of GhTIFY10A Delayed Leaf Senescence in Transgenic Arabidopsis Plants

**Figure 5:**
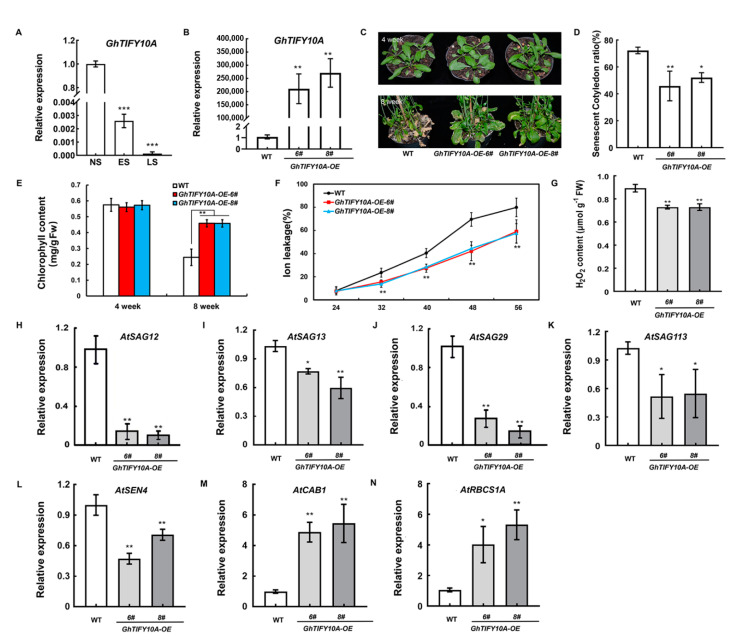
Overexpression of *GhTIFY10A* delayed leaf senescence in transgenic *Arabidopsis* plants. (**A**) RT-qPCR analysis of *GhTIFY10A* transcript levels in cotton wild-type leaves at different developmental stages. Transcript levels of *GhWRKY33* in NS leaves were arbitrarily set to 1. (**B**) Transcript levels of *GhTIFY10A* in *Arabidopsis* transgenic plants. (**C**) Phenotypic characteristics of WT and transgenic plants grown for four and eight weeks, respectively. Plants were grown in a growth chamber at 22 °C under a 16 h light/8 h dark photoperiod. The natural senescence phenotype was observed from about 72 plants for each line, and representative plants were photographed. (**D**) Relative senescent cotyledon ratio of WT and *GhTIFY10A-OE* plants grown for four weeks. (**E**) Chlorophyll content in detached rosette leaves of WT and *GhTIFY10A-OE* plants grown for four and eight weeks, respectively. FW, fresh weight. (**F**) Membrane ion leakage of WT and *GhTIFY10A*-OE plants at the indicated leaf age. Leaves three to four from 12 to 15 plants of each genotype (approximately 26 leaves) were harvested and pooled. (**G**) H_2_O_2_ content in rosette leaves of WT and *GhTIFY10A-OE* plants grown for eight weeks. (**H**–**N**) RT-qPCR analysis of transcript levels of senescence marker genes in leaves of WT and *GhTIFY10A*-*OE* plants in eight weeks. Data from three biological replicates were analyzed by ANOVA, and asterisks indicate significant differences compared with WT (* *p* < 0.05; ** *p* < 0.01; *** *p* < 0.001).

Given that GhWRKY33 and GhTIFY10A physically interact, and *GhWRKY33* transgenic plants have a delayed senescence phenotype, we speculate that *GhTIFY10A* may also play a role in leaf senescence. We first determined the expression of *GhTIFY10A* in cotton senescing leaf. As shown in Figure 5A, similar to *GhWRKY33*, the expression level of *GhTIFY10A* was also dramatically lower in early senescent leaves (ES) and was further repressed in late senescent leaves (LS) than that of non-senescent leaves (NS). These results showed that *GhTIFY10A* was repressed during cotton leaf senescence, indicating that *GhTIFY10A* may also play a role in leaf senescence.

To further confirm the role of *GhTIFY10A* in leaf senescence, four homozygous transgenic *Arabidopsis* lines (T_3_) heterologously expressing *GhTIFY10A* under the control of the CaMV 35S promoter (*GhTIFY10A-OE-6*#, *GhTIFY10A-OE-8*#, *GhTIFY10A-OE-10*# and *GhTIFY10A-OE-11*#) were used for further study Figure 5B and Figure S6A). Interestingly, ageing-triggered leaf senescence was clearly delayed in *GhTIFY10A*-overexpressing plants compared with wild-type (Figure 5C,D and Figure S6B). The transgenic plants also displayed a significantly elevated chlorophyll content, lower ion leakage, significantly reduced H_2_O_2_ content, and enhanced the expression of the photosynthetic genes (*AtCAB1* and *AtRBCS1A*) but reduced expression of several *SAG*s than wild-type plants (Figure 5E–N). Thus, constitutive overexpression of *GhTIFY10A* in *Arabidopsis* led to delayed leaf senescence.

### 3.6. Both GhWRKY33 and GhTIFY10A Negatively Regulate JA-Induced Leaf Senescence

**Figure 6:**
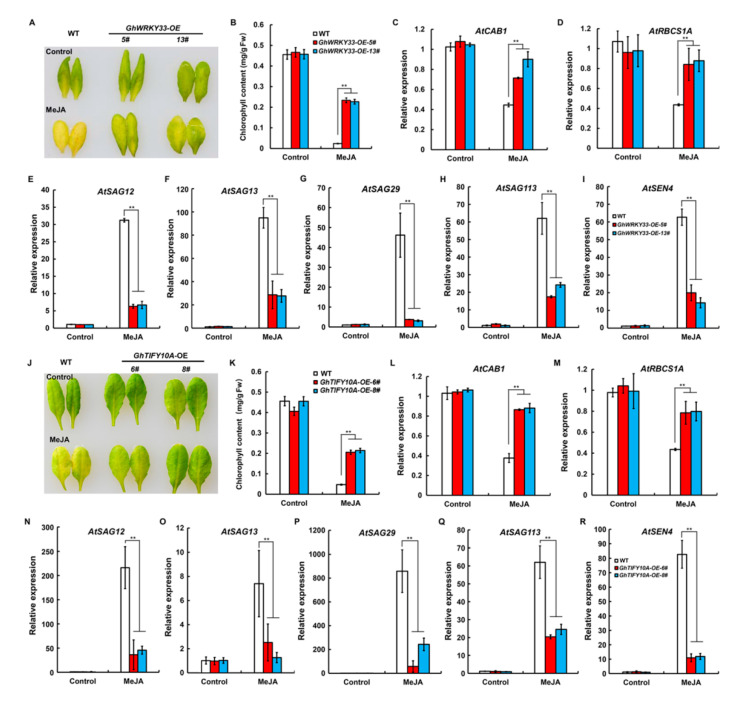
Both *GhWRKY33* and *GhTIFY10A* delayed JA-induced leaf senescence. (**A**,**J**) Phenotypes of indicated genotypes. The wild-type leaves showed a more severe JA-induced senescence phenotype than both *GhWRKY33* and *GhTIFY10A* overexpression lines upon 100 µM MeJA treatment. (**B**,**K**) Chlorophyll content in detached full-grown rosette leaves of the indicated genotypes. (**C**–**I**,**L**–**R**) Transcript levels of *AtSAGs, AtCAB1,* and *AtRBCS1A* in the indicated genotypes. Date from three biological replicates were analyzed by ANOVA, and asterisks indicate significant differences compared with WT (** *p* < 0.01).

Given that GhWRKY33 and GhTIFY10A physically interact and both negatively regulate leaf senescence, we speculated that they might also participate in the regulation of JA-induced leaf senescence. Interestingly, both *GhWRKY33* and *GhTIFY10A* are induced by MeJA treatment, implying their possible involvement in JA-induced leaf senescence (Figure S7). Then, the detached leaves of *GhWRKY33-OE* lines, *GhTIFY10A-OE* lines, and WT were used for JA-induced leaf senescence assays. After MeJA treatment, compared with WT, *GhWRKY33-OE* and *GhTIFY10A-OE* leaves showed less severe yellowing (Figure 6A,J). Furthermore, the measurement of chlorophyll content also showed that chlorophyll was lost more quickly in the leaves of WT than in the *GhWRKY33-OE* and *GhTIFY10A-OE* lines upon MeJA treatment (Figure 6B,K). Consistently, after treatment with MeJA, the transgenic plants also showed reduced expression of several *SAGs* but the enhanced expression of the photosynthetic genes (*AtCAB1* and *AtRBCS1A*) than wild-type plants (Figure 6C–I,L–R).These results suggested that both *GhWRKY33* and *GhTIFY10A* play negative roles in JA-induced leaf senescence.

### 3.7. GhTIFY10A and GhWRKY33 Acts Synergistically to Suppress Both AtSAG12 and Ghcysp Expression

**Figure 7:**
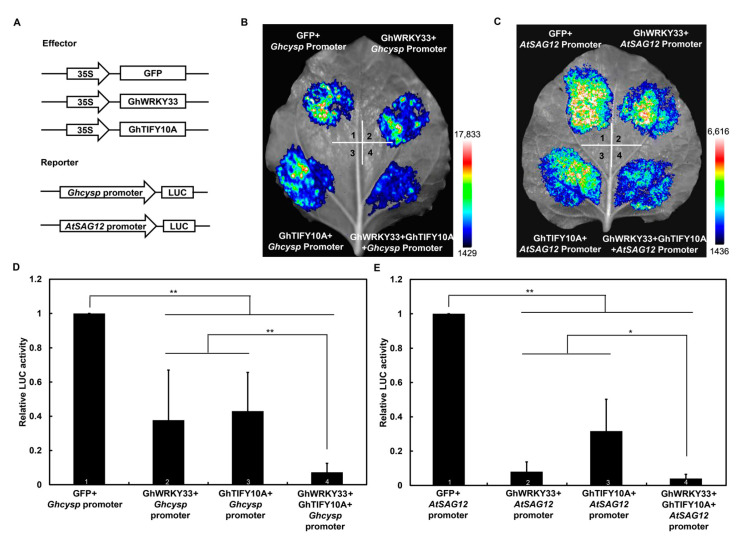
GhWRKY33 and GhTIFY10A synergistically suppress the expression of *SAG*s in the dual-luciferase reporter assay. (**A**) Sketch of the effector and reporter constructs. The *GhWRKY33* and *GhTIFY10A* were cloned into the effector vector *pGreenII 62-SK*, and the promoters of *Ghcysp* and *AtSAG12* were cloned into the reporter vector *pGreenII 0800-LUC*. (**B**–**E**) GhWRKY33 and GhTIFY10A synergistically suppress *Ghcysp* and *AtSAG12* expression in transient dual-luciferase reporter systems. The relative LUC activity of the combination of *pGreenII-62-SK* empty vector and *Ghcysp* and *AtSAG12* promoter were set as 1. Data from six biological replicates were analyzed by ANOVA, and asterisks indicate significant differences (* *p* < 0.05; ** *p* < 0.01).

Having demonstrated that GhTIFY10A physically interacts with GhWRKY33, we speculated that it might affect the transcriptional function of *GhWRKY33*. To test this possibility, a transient dual-luciferase assay was performed in tobacco leaves to elucidate the functional role of *GhWRKY33* and *GhTIFY10A* in regulating the expression of *SAGs in vivo*. The experiment was conducted using a double reporter plasmid, *pGreenII0800-LUC*, containing the LUC luciferase driven by the *AtSAG12* and *Ghcysp* promoters. In addition, the assay includes effecters plasmid, *pGreenII62-SK*, expressing the *GhWRKY33* and *GhTIFY10A* (Figure 7A). The results showed that the expression of *GhWRKY33* or *GhTIFY10A* resulted in reduced LUC signals compared with the reporters alone. More importantly, coexpression of *GhWRKY33* with *GhTIFY10A* further reduced the LUC signals compared with the expression of *GhWRKY33* or *GhTIFY10A* alone (Figure 7B–E).These results support the hypothesis that GhWRKY33 and GhTIFY10A act synergistically to suppress both *AtSAG12* and *Ghcysp* expression.

## 4. Discussion

To increase survival and fitness in their given ecological niches, plants always trigger leaf senescence to relocate mobile nutrients and energy from aging leaves to reproducing seeds [30]. Senescence is the final stage of leaf development and is tightly controlled by a sophisticated transcriptional regulatory network in which transcription factors are widely participating. Notably, numerous WRKY transcription factors show strong expression in senescing leaves, implying their potential involvement in senescence-associated transcriptional reprogramming [12]. The functional studies identified several WRKY TFs as critical regulators in leaf senescence, but their specific biological functions in this process remain elucidated. In particular, the functional elucidation of most *WRKY* genes in cotton represents a major challenge.

Interestingly, recent studies have demonstrated that WRKY proteins often function as key components of various phytohormone-mediated leaf senescence. For example, AtWRKY45 interacts with DELLA protein RGL1 to positively regulates GA-mediated leaf senescence, while AtWRKY75 directly promotes *SA INDUCTIONDEFICIENT2* (*SID*2) expression to promote SA production and finally accelerates leaf senescence [4,31]. Recently, OsWRKY53 was shown to accelerate leaf senescence through the promotion of ABA accumulation, and AtWRKY75 can also play a role in ABA-mediated leaf senescence [21,32]. Although several cotton *WRKY* members, including *GbWRKY27*, *GhWRKY42*, and *GhWRKY91*, have been shown to play important roles in leaf senescence [15,16,17], it is still unclear whether they can regulate leaf senescence through interaction with certain phytohormones. Here, we provide further evidence to reveal that GhWRKY33 may function as a new component that regulates both ageing and JA-mediated leaf senescence.

We found that *GhWRKY33* is repressed in senescing leaves and transgenic *Arabidopsis* plants overexpressing *GhWRKY33* delayed both ageing and JA-triggered leaf senescence (Figure 1, Figure 2 and Figure 6). WRKY TFs specifically bind to the W-boxes of the promoters of their target genes and activate/repress their expression to regulate a variety of physiological processes, such as plant growth and development, defense response, and leaf senescence [4,14,29,33]. In our study, GhWRKY33 was also able to bind to the promoters of both *AtSGA12* and *Ghcysp*, and inhibit their expression. Taken together, these results imply that *GhWRKY33* may function as a negative regulator to modulate both age and JA-mediated leaf senescence. A recent study also revealed that *GhWRKY33* can function as a negative regulator to mediate plant response to drought stress [28]. Thus, GhWRKY33 may mainly function as a repressor to modulate both plant growth and stress responses. However, it is still interesting to determine whether GhWRKY33 can function as an activator in certain physiological processes.

As a lipid-derived plant hormone, jasmonates (JAs) were revealed to play crucial roles in both plant defense responses and various developmental processes [34]. Studies have demonstrated that the JA signal is perceived by the F-box protein CORONATINE INSENSITIVE1 (COI1), which subsequently recruits the JASMONATEZIM-DOMAIN (JAZ) proteins for ubiquitination and degradation [35,36,37], leading to the release and activation of various downstream transcription factors that modulate corresponding JA responses.

In *Arabidopsis*, several critical transcription factors have been identified as direct targets of JAZ proteins. For example, bHLH subgroup IIIe transcription factors (MYC2, AtMYC3, and AtMYC4), essential components of WD-repeat/bHLH/MYB transcriptional complexes (TRANSPARENT TESTA 8 [TT8], GLABRA 3 [GL3], ENHANCER OF GLABRA 3 [EGL3], R2R3 MYB transcription factors [MYB75 and Glabra1]), ROOT HAIR DEFECTIVE 6 [RHD6]), and APETALA2 transcription factors (TARGET OF EAT1 [TOE1] and TOE2) all can function as direct targets of JAZ protein to regulate JA-mediated plant defense, anthocyanin accumulation, trichome initiation, root hair growth, and flowering respectively [38,39,40,41,42]. Recently, AtWRKY57 and AtWRKY75 were also identified as targets of JAZs to modulate JA-mediated leaf senescence and plant defense against necrotrophs, respectively [3,43]. Until now, it remains unclear whether certain GhJAZs can interact with GhWRKYs to regulate leaf senescence. Here, we provide evidence that GhWRKY33 may negatively regulate leaf senescence through the JA pathway.

About 50 *GhTIFY*s members were recently identified in upland cotton (*G. hirsutum*) [44], and their functional elucidation remains a big challenge. Furthermore, there is also no report about their involvement in leaf senescence. In this study, we reveal that *GhTIFY10A* may function as a negative regulator to modulate both ageing and JA-triggered leaf senescence. Similar to *GhWRKY33*, *GhTIFY10A* was repressed in cotton senescing leaves, and transgenic *Arabidopsis* plants overexpressing *GhTIFY10A* delayed both ageing and JA-triggered leaf senescence (Figure 5 and Figure 6). The yeast two-hybrid (Y2H) and firefly luciferase complementation imaging (LUC) assays showed that GhWRKY33 could interact with GhTIFY10A (Figure 4). Further analysis using the LUC assay demonstrated that GhTIFY10A enhances the transrepression activity of GhWRKY33 and subsequently synergistically repressed the expression of *AtSGA12* and *Ghcysp*, and finally, negatively modulated both ageing and JA-mediated leaf senescence (Figure 7). Thus, our results indicated that JAZ-targeted GhWRKY33 may negatively modulate plant leaf senescence by directly targeting senescence-associated genes.

The early and abnormal senescence shortens plant lifespan and decreases crop yield and quality. Therefore, it is essential to study the underlying mechanisms and signaling pathways involved in leaf senescence, which will be highly useful for crop genetic breeding. Here, we demonstrate the molecular mechanisms underlying the regulation of both ageing and JA-triggered leaf senescence by GhWRKY33. They indicate that GhWRKY33 may function as a novel component of the cotton senescence regulatory network in both ageing and JA-mediated leaf senescence via interaction with GhTIFYs. Thus, our results represent a new insight into the roles of GhWRKY proteins in senescence-associated signaling and transcriptional reprogramming, and also lay a foundation for further functional studies on cotton leaf senescence.

## Data Availability

The datasets generated during and/or analysed during the currentstudy are available from the corresponding author on a reasonable request.

## Supplementary Materials

The following supporting information can be downloaded at: https://www.mdpi.com/article/10.3390/cells11152328/s1, Figure S1: *GhWRKY33* delayed leaf senescence in transgenic *Arabidopsis* plants; Figure S2: The expression level of *GhWRKY33* in leaves of different senescence stages of the cotton variety TX2094 and Lumianyan 28; Figure S3: GhWRKY33 suppressed the expression of both *AtSAG12* and *Ghgysp*; Figure S4: GhWRKY33 physically interacts with GhTIFY6B; Figure S5: Analysis of conservative motif TIFY and Jas in GhTIFY10A; Figure S6: *GhTIFY10A* delayed leaf senescence in transgenic *Arabidopsis* plants; Figure S7: Both *GhWRKY33* and *GhTIFY10A* were induced by MeJA treatment; Table S1: Primers used in this study.
