# Supplementary material for: GhWRKY33 Interacts with GhTIFY10A to Synergistically Modulate Both Ageing and JA-Mediated Leaf Senescence in Arabidopsis
Source: Cells. 2022 Jul 29;11(15):2328. doi: 10.3390/cells11152328 (PMC9367327; doi:10.3390/cells11152328)
Supplement: Supplementary file 1 [file cells-11-02328-s001.zip › cells-1728604-supplemtary.pdf]

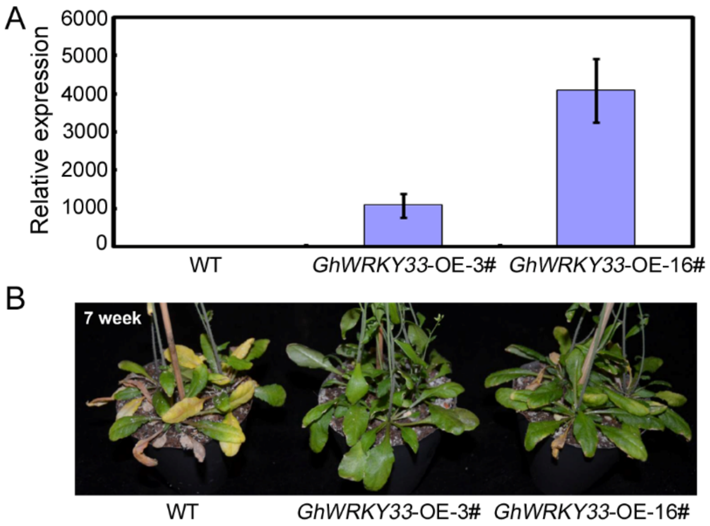

Figure S1. *GhWRKY33* delayed leaf senescence in transgenic *Arabidopsis* plants.

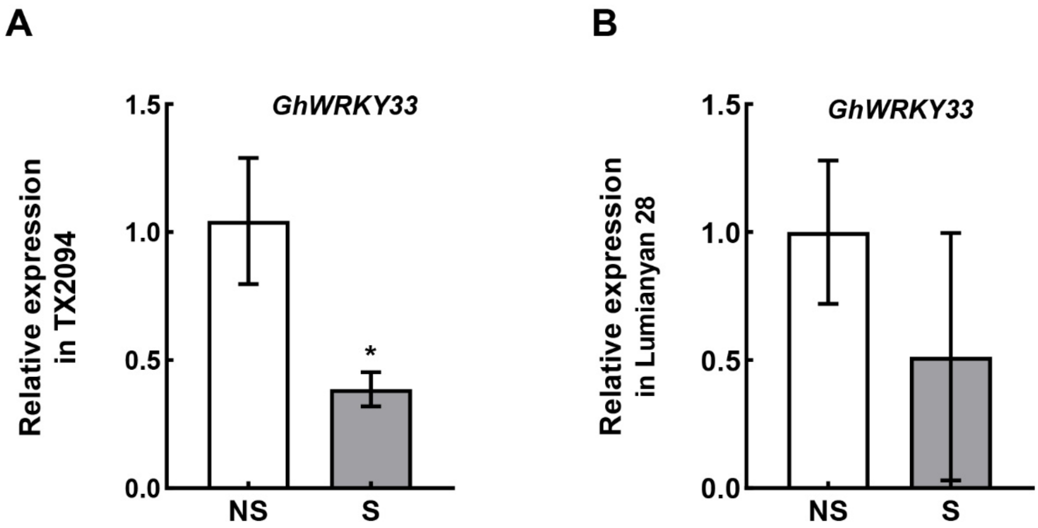

Figure S2. The expression level of *GhWRKY33* in leaves of different senescence stages of the cotton variety TX2094 and Lumianyan 28.

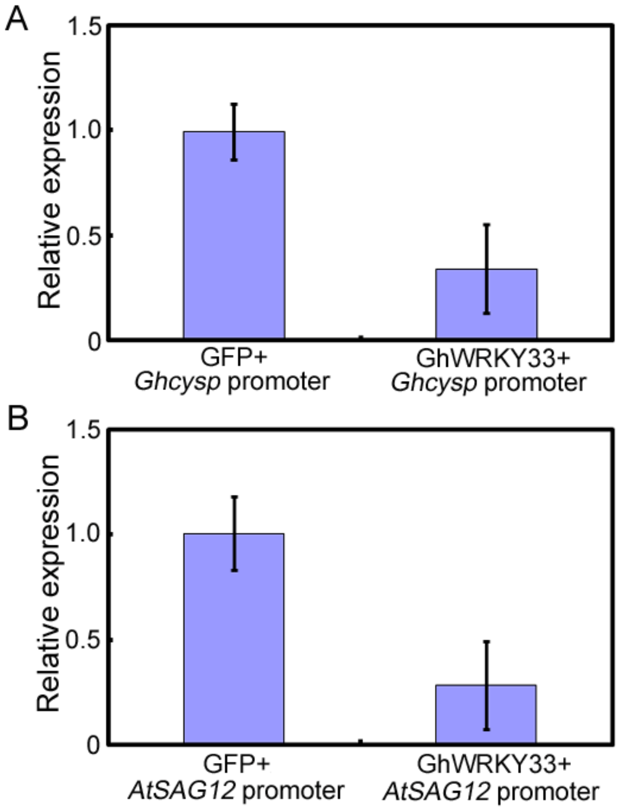

Figure S3. GhWRKY33 suppressed the expression of both *AtSAG12* and *Ghgysp*.

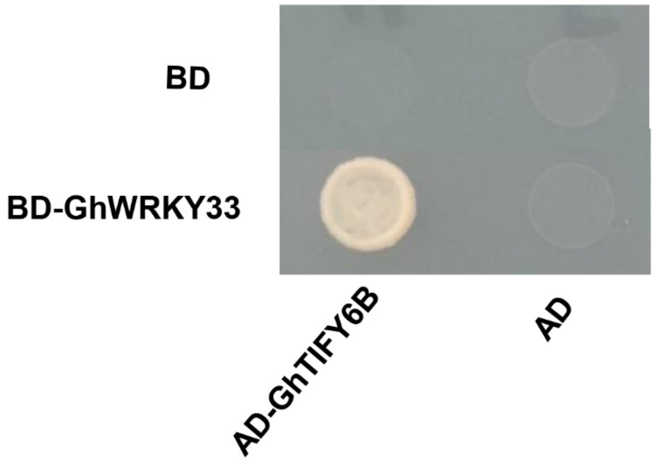

Figure S4. GhWRKY33 physically interacts with GhTIFY6B.

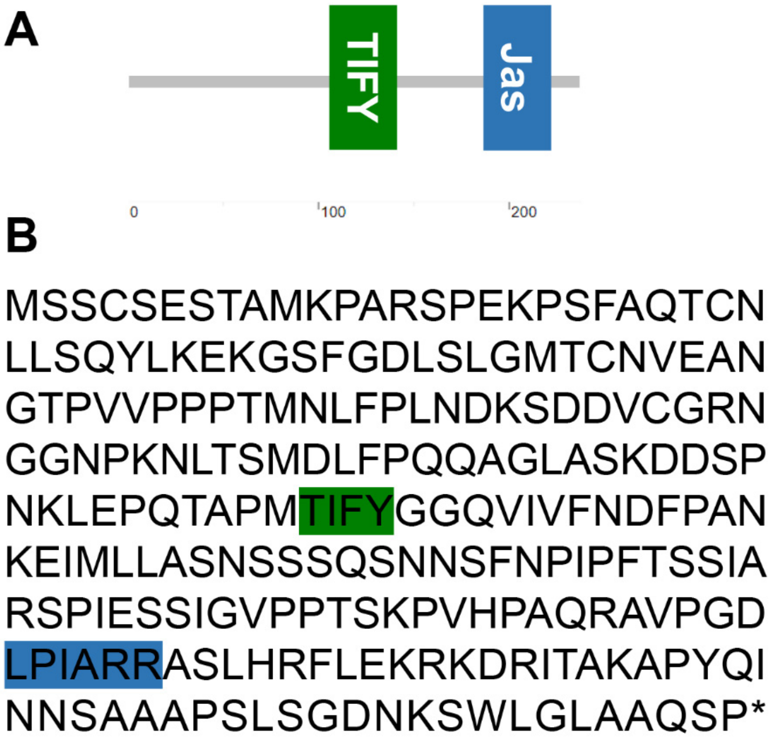

B

MSSCSESTAMKPARSPEKPSFAQTCN
LLSQYLKEKGSFGDLSLGMTCNVEAN
GTPVVPPPTMNLFPLNDKSDDVCGRN
GGNPKNLTSMDLFPQQAGLASKDDSP
NKLEPQTAPMTIFYGGQVIVFNDFPAN
KEIMLLASNSSSQSNNSFNPIPFTSSIA
RSPIESSIGVPPTSKPVHPAQRAVPGD
LPIARRASLHRFLEKRKDRITAKAPYQI
NNSAAAPSLSGDNKSWLGLAAQSP*

Figure S5. Analysis of conservative motif TIFY and Jas in GhTIFY10A.

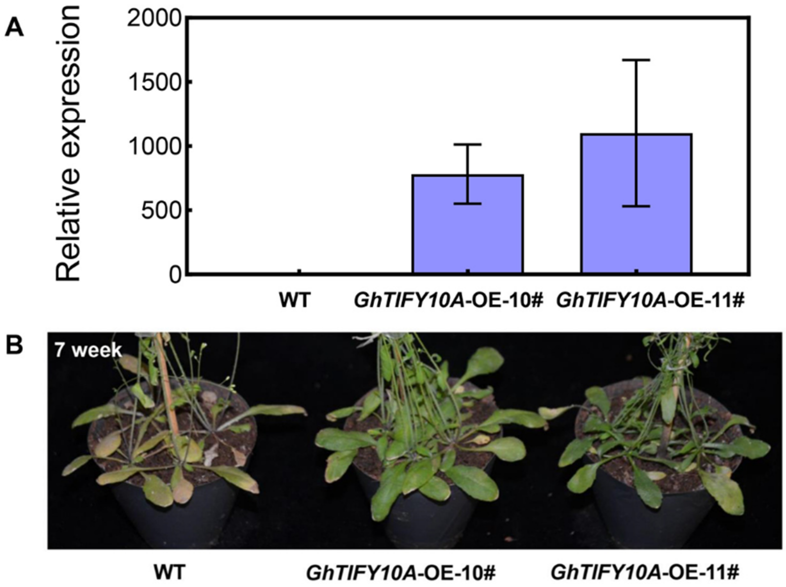

Figure S6. *GhTIFY10A* delayed leaf senescence in transgenic *Arabidopsis* plants.

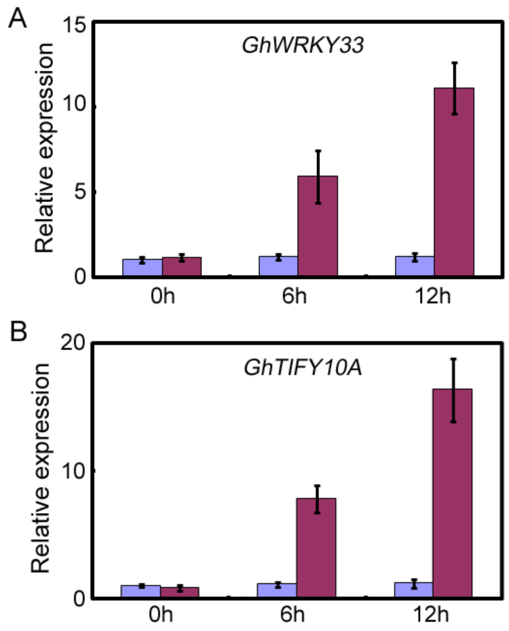

Figure S7. Both *GhWRKY33* and *GhTIFY10A*were induced by MeJA treatment.
